# Temporal and spatial patterns of bovine *Escherichia coli *O157 prevalence and comparison of temporal changes in the patterns of phage types associated with bovine shedding and human *E. coli *O157 cases in Scotland between 1998-2000 and 2002-2004

**DOI:** 10.1186/1471-2180-9-276

**Published:** 2009-12-29

**Authors:** Michael C Pearce, Margo E Chase-Topping, Iain J McKendrick, Dominic J Mellor, Mary E Locking, Lesley Allison, Helen E Ternent, Louise Matthews, Hazel I Knight, Alastair W Smith, Barti A Synge, William Reilly, J Christopher Low, Stuart WJ Reid, George J Gunn, Mark EJ Woolhouse

**Affiliations:** 1 8 Stour Court, Sandwich, Kent, CT13 9FY, UK; 2 Centre for Infectious Diseases, University of Edinburgh, Kings Buildings, Edinburgh, EH9 3J5, UK; 3 Biomathematics and Statistics Scotland (BioSS), James Clerk Maxwell Building, King's Buildings, Edinburgh EH9 3JZ, UK; 4 Comparative Epidemiology and Informatics, Department of Veterinary Clinical Studies, University of Glasgow Veterinary School, 464 Bearsden Rd, Glasgow G61 1QH, UK; 5 Health Protection Scotland, Clifton House, Clifton Place, Glasgow G3 7LN, UK; 6 Scottish E. coli O157/VTEC Reference Laboratory, Western General Hospital, Edinburgh, UK; 7 Scottish Agricultural College, Animal Health Group, Research Division, King's Buildings, West Mains Road, Edinburgh, EH9 3JG, UK

## Abstract

**Background:**

*Escherichia coli *O157 is an important cause of acute diarrhoea, haemorrhagic colitis and, especially in children, haemolytic uraemic syndrome (HUS). Incidence rates for human *E. coli *O157 infection in Scotland are higher than most other United Kingdom, European and North American countries. Cattle are considered the main reservoir for *E. coli *O157. Significant associations between livestock related exposures and human infection have been identified in a number of studies.

**Results:**

***Animal Studies: ***There were no statistically significant differences (*P *= 0.831) in the mean farm-level prevalence between the two studies (SEERAD: 0.218 (95%CI: 0.141-0.32); IPRAVE: 0.205 (95%CI: 0.135-0.296)). However, the mean pat-level prevalence decreased from 0.089 (95%CI: 0.075-0.105) to 0.040 (95%CI: 0.028-0.053) between the SEERAD and IPRAVE studies respectively (*P *< 0.001). Highly significant (*P *< 0.001) reductions in mean pat-level prevalence were also observed in the spring, in the North East and Central Scotland, and in the shedding of phage type (PT) 21/28. ***Human Cases: ***Contrasting the same time periods, there was a decline in the overall comparative annual reported incidence of human cases as well as in all the major PT groups except 'Other' PTs. For both cattle and humans, the predominant phage type between 1998 and 2004 was PT21/28 comprising over 50% of the positive cattle isolates and reported human cases respectively. The proportion of PT32, however, was represented by few (<5%) of reported human cases despite comprising over 10% of cattle isolates. Across the two studies there were differences in the proportion of PTs 21/28, 32 and 'Other' PTs in both cattle isolates and reported human cases; however, only differences in the cattle isolates were statistically significant (*P *= 0.002).

**Conclusion:**

There was no significant decrease in the mean farm-level prevalence of *E. coli *O157 between 1998 and 2004 in Scotland, despite significant declines in mean pat-level prevalence. Although there were declines in the number of human cases between the two study periods, there is no statistically significant evidence that the overall rate (per 100,000 population) of human *E. coli *O157 infections in Scotland over the last 10 years has altered. Comparable patterns in the distribution of PTs 21/28 and 32 between cattle and humans support a hypothesized link between the bovine reservoir and human infections. This emphasizes the need to apply and improve methods to reduce bovine shedding of *E. coli *O157 in Scotland where rates appear higher in both cattle and human populations, than in other countries.

## Background

In the last 25 years, *Escherichia coli *serogroup O157 (*E. coli *O157) has become an important cause of severe gastrointestinal illness in westernised countries, warranting substantial public health concern. Clinical signs range from mild diarrhoea to haemorrhagic colitis and haemolytic uraemic syndrome (HUS) which may result in death [1]. HUS usually occurs in young children and is the major cause of acute renal failure in children in western countries [2]. Clinical surveillance in Scotland has shown that over 90% of HUS cases are associated with *E. coli *O157 infection [3]; similar observations have been made in other countries [4-6]. Cattle are the main reservoir for *E. coli *O157 [7], and play a major role in the epidemiology of human infections [8]. Visits to farms, contact with animal excreta and recreational use of animal pasture have all been identified as significant risk factors for sporadic human infections [9-12]. Spatial analyses suggest that human incidence is positively associated with indicators such as livestock density and the ratio of cattle to human population, although the relationship appears complex [13-16].

Considerable effort has been made to determine the prevalence of *E. coli *O157 in cattle worldwide (Brazil: [17], Canada: [18], Denmark: [19], England: [20], Iran: [21], Netherlands: [22]; Norway: [23], Spain: [24], Sweden: [25], United States: [26]). Estimates of prevalence range from 0 to 71% of animals and 0 to 100% of herds [27]. Two of the world's largest surveys of animal *E. coli *O157 prevalence were conducted in the past decade in Scotland. The first [28] estimated herd-level and animal-level prevalence for 952 farms throughout Scotland in a study funded by the Scottish Executive Environment and Rural Affairs Department (SEERAD) conducted from March 1998 to May 2000. Since then a second survey, funded by the Wellcome Foundation International Partnership Research Award in Veterinary Epidemiology (IPRAVE) was conducted on a subsample of the 952 SEERAD farms, from February 2002 to February 2004. Data from the SEERAD and IPRAVE studies are presented in this paper.

In Scotland, the first reported cases of human *E. coli *O157 infection were identified in 1984. Currently, Health Protection Scotland (HPS) conducts active, population based enhanced surveillance in close collaboration with the Scottish *E. coli *O157/VTEC Reference laboratory (SERL) [29]. Over the 10 year period 1998-2007, an annual average of 221 culture positive cases has been reported to HPS, which is an average annual rate of 4.28 cases per 100,000 population [30]. Rates in Scotland are generally higher than in most other United Kingdom, European and North American countries [30-33].

A recent publication proposed a specific mechanism for the link between human infection and livestock carriage of *E. coli *O157 [34] which involved a subset of shedding animals known as super-shedders. Super-shedders are individuals who for a period yield more infectious organisms (here *E. coli *O157) than typical individuals of the same host species [34]. Shedding high concentrations of *E. coli *O157 has been proposed as a major contributor to cattle-to-cattle transmission [34-36] and possibly cattle-to-human transmission. Although little is known about super-shedders it has been shown that they have been associated with the presence of phage type (PT) 21/28 whereas non super-shedders are more likely to be associated with PT32 [37]. Recent evidence has shown PT21/28 to be associated with higher transmission in livestock when compared to PT32 [38]. PT21/28 is the most predominant phage type in both cattle [37] and human cases [39] whereas PT32 is a common phage type in cattle only [37]. In humans, PT21/28 is of particular concern because of its association with more severe morbidity. In the UK and Ireland (1997-2001), the mean risk of developing diarrhoea-associated HUS was significantly higher in children in Scotland infected with PT21/28 compared with other phage types [40]. Phage type 21/28 strains possess the *vtx*_*2 *_gene alone, which appears to be associated with more serious health outcomes [41].

The objectives of this study were three-fold. First, to calculate the mean prevalence of *E. coli *O157 in cattle using the data from both the SEERAD (1998-2000) and IPRAVE (2002-2004) surveys. Second, to examine temporal patterns in the overall as well as regional, seasonal and phage type specific prevalence of bovine shedding. Third, to examine the incidence levels and relative proportions of common phage types associated with human cases over the same periods and the proportion of phage types PT21/28 and PT32 in bovine isolates and human cases, for evidence of any epidemiological link between the two.

## Methods

### Animal Prevalence Studies

#### Livestock Sampling Design

Two surveys of Scottish store and finishing cattle were conducted: the first from March 1998 to May 2000, the second from February 2002 to February 2004. The first study was funded by the Scottish Executive Environment and Rural Affairs Department (SEERAD); the second by a Wellcome Foundation International Partnership Research Award in Veterinary Epidemiology (IPRAVE). Details on the methodology of both surveys have been published elsewhere [28,37,42], however, a brief outline is given below.

**Figure 1:**
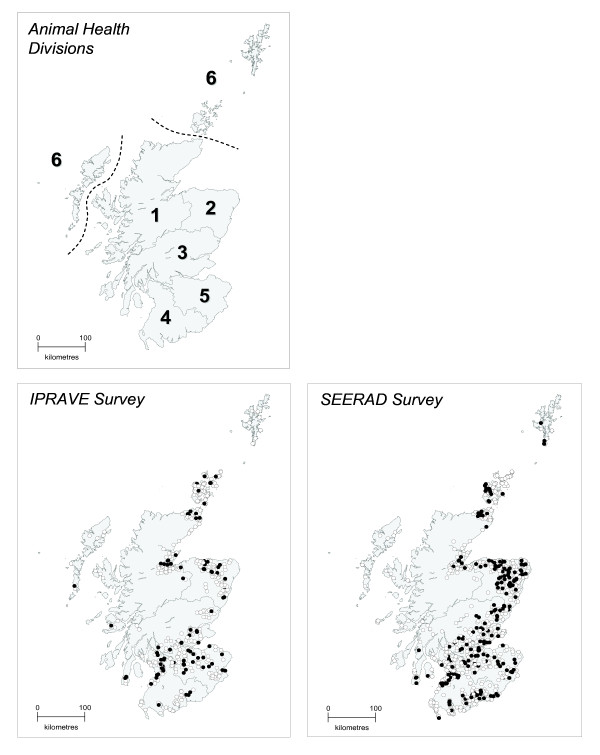
**Location of State Veterinary Service animal health divisions and sampled farms with store and finishing cattle**. Animal health divisions: 1, Highlands; 2, North East; 3, Central; 4, South West; 5, South East; 6, Islands. Open circle, farms where no *E. coli *O157 shedding was detected; closed circle, farms where *E. coli *O157 shedding was detected. This work is based on data provided with the support of the ESRC and JISC, and uses boundary material which is copyright of the Crown and the Post Office. Source: the 1991 Census, Crown Copyright. ESRC purchase.

In 1998, SEERAD provided the Scottish Agricultural College (SAC) with a list comprising 3,111 farms with cattle, randomly selected from 1997 Scottish Agricultural and Horticultural Census data. For the SEERAD survey, 952 farms across the 6 state animal health divisions (AHDs) (Highland, Islands, North East, Central, South East, South West) (Figure 1) were randomly selected and surveyed [28]. Owners or managers of 925 of these 952 farms consented to an additional sampling visit and these 925 farms were used as the sampling frame for the second survey (IPRAVE). Within the sampling frame for the IPRAVE survey there were insufficient farms to adequately represent two state animal health divisions: Highland and Islands. Additional farms (n = 34) for these two AHDs were recruited by random selection from the remainder of 3,111 farms not sampled in the SEERAD survey. In total, 481 farms were sampled for the IPRAVE survey, 447 of which had been previously sampled in the SEERAD survey. Instead of randomly sampling farms within each AHD, the IPRAVE study used a stratified sampling plan to select farms to sample [42]. This was done to ensure that similar numbers were included from each region and that regions were sampled evenly over time.

Both surveys preferentially sampled cattle groups composed only of store (i.e. weaned cattle before finishing for slaughter) or finishing cattle closest to sale or slaughter. If such groups did not exist, one or more mixed groups with store or finishing cattle closest to sale or slaughter were sampled. From each group fresh faecal pats were sampled. The number of faecal pats tested in each group was determined from the number of cattle in the group using a prescribed sampling schedule. For the SEERAD survey, sufficient numbers of faecal pats were tested to ensure prospectively an 80% chance of sampling at least one positive pat if there was a shedding prevalence of at least 2% within the group [28]. Based on results from the SEERAD survey, in the IPRAVE survey, it was assumed that, on average, 8% of the animals in positive groups would be shedding, with shedding distributed as seen in the SEERAD survey [28]. For each IPRAVE group, sufficient fresh pat samples were taken to ensure prospectively a mean 90% probability of detecting shedding of *E. coli *O157 if at least one shedding animal was indeed present.

Samples were collected from freshly voided faecal pats, refrigerated at 5°C as soon as possible and processed within 48 hours of collection. No direct animal sampling was involved and the study was not regulated by The Animals (Scientific Procedures) Act 1986. At present the SEERAD and IPRAVE data are not available on open-access databases, however, requests for data can be made though the corresponding author.

#### Immunomagnetic Separation (IMS) and Phage Typing of Livestock samples

Within 48 hours of sampling, one gram of faeces from each sample was tested for the presence of *E. coli *O157 as previously described [43]. Following IMS, one *E. coli *O157 isolate from each faecal sample was submitted to the Scottish *E. coli *O157/VTEC Reference Laboratory (SERL) for phage typing [44], and tested for the presence of genes encoding the virulence factors verocytotoxin 1 (*vtx*_*1*_), verocytotoxin 2 (*vtx*_*2*_) and intimin (*eae*) using multiplex PCR [45,46].

### Human Case Identification, Data Collection and Phage Typing

Health Protection Scotland (HPS) receives reports of human cases of *E. coli *O157 infection from SERL and from diagnostic laboratories throughout Scotland. Diagnostic laboratories submit samples (isolates, faeces and sera) to SERL for further testing in line with Scottish guidance [47]. Using a series of phenotypic and genotypic tests, SERL confirms the identity of submitted isolates of *E. coli *O157, or identifies and isolates *E. coli *O157 from submitted faecal samples [48]. SERL also types all isolated organisms using a hierarchical array of methods including phage typing, polymerase chain reaction (PCR) and pulse-field gel electrophoresis (PFGE). The results of phage and verotoxin typing undertaken by SERL are also reported to HPS.

Since establishing enhanced surveillance in 1999, HPS has compiled a standard dataset for every case, in which microbiological data shared by SERL is a crucial component [29]. A case is defined as one person-infection-episode, with microbiological confirmation of infection, defined as culture positive i.e. isolates of *E. coli *O157 cultured from faeces. Although HPS enhanced surveillance also includes cases identified by SERL by detection of antibodies to *E. coli *O157 in serum, these serum positive cases were excluded from data entered into this study as they by definition had no available phage type results.

HPS integrates laboratory data including SERL typing results, with epidemiological details from local investigators (primarily public health). These include clinical and exposure details, including travel. Prior to 1999, the number of cases that might potentially have acquired infection outside the UK could only be estimated according to whether non-UK countries were noted on laboratory forms; details of whether travel occurred before, during or after the incubation period were not available to HPS. Since 1999, enhanced surveillance at HPS has enabled more accurate differentiation of imported cases defined as likely to have acquired infection outside the UK, based on examination of travel, clinical and exposure histories provided by local investigators [29].

Data on culture-positive, indigenous human cases with known phage type results identified by SERL, for the period March 1998 to May 2000 (n = 793 days) and February 2002 to February 2004 (n = 734 days), were therefore entered into this study by HPS, in collaboration with SERL.

### Statistical Analysis

#### Animal Studies - Prevalence of *E. coli *O157

The SAS v9.1.3 package (SAS Institute, Cary, NC, USA) was used to fit generalised linear mixed models (GLMMs), to generate bootstrap-based estimates of key parameters and to carry out non-parametric statistical testing. The Excel 2000 package (Microsoft Corporation) was used to implement a Latin hypercube sampling algorithm to convert results from the GLMMs into prevalence, taking into account the influence of random effects [49] and to assess the group sensitivity of the two sampling regimens. Seasons were defined as: winter, December, January and February; spring, March, April, and May; summer, June, July and August; and winter, September, October and November. Statistical significance of pairwise comparisons was determined using Students t-test.

#### Farm-level data analysis

The mean percentage of farms with shedding cattle was estimated using GLMMs [50], fitting models with Bernoulli response terms and a logit link function. A farm was classed as positive if at least one animal was identified as shedding. Farm cluster and/or farm were fitted as random effects depending on the sampling design of the program. Including AHD and season as fixed effects, GLMMs were used to determine the impact of AHD and season on the proportion of farms with shedding cattle in each AHD and season. Confidence intervals for means were derived by reweighting output from the appropriate GLMM, taking into account the influence of random effects [49]. Latin hypercube sampling of the observed non-zero prevalences and sample sizes was used to provide inputs to a simple probabilistic calculation, assuming sampling with replacement, of mean estimates of the sensitivity of the sampling procedures in identifying positive groups.

#### Pat-level data analysis

For both the SEERAD and IPRAVE surveys, sampling distributions of the overall mean prevalence of shedding, overall mean shedding prevalence by specific phage type, and mean shedding prevalence within AHD or seasonal subsets were generated using bootstrap sampling with 10,000 iterations. In each iteration, farms and pats from each farm were sampled from the overall data or respective AHD or seasonal subsets arising from the original surveys. The same number of pats sampled in the original surveys was sampled using the sampling procedure used in the original surveys, but with replacement both at the farm and pat strata. The mean and upper and lower confidence limits of the mean shedding prevalence were derived from the respective bootstrap distributions. These calculations make no adjustment for the sensitivity and specificity of the assay.

### Human Data Analysis--Incidence of Common Phage Types

The number of human cases entered into the study and the duration of the surveys were used to calculate the comparative incidence of human cases. This was then expressed as an equivalent annual figure. Incidence was calculated as the number of human cases with each of the more common phage types (PT2, PT21/28, PT32, PT4, PT8) and 'Other' PTs (comprising PT34, PT14, PT31, PT33, PT54, isolates having an RDNC phage type, where the phages react but do not conform to a known pattern, and Untypeable) reported to HPS over the time periods equivalent to the SEERAD and IPRAVE surveys.

### Comparison of Phage Types from Cattle and Human Cases

The overall temporal pattern of the most common phage types ie PT2, PT21/28, PT32, PT4, PT8 and 'Other' PTs (comprising PT34, PT14, PT31, PT33, PT49, PT54, PT24, RDNC and Untypeable) were examined for human cases and cattle isolates using the Cochran Mantel Haenzel (CMH) Test (unordered stratified RxC) (StatXact v.8, Cytel Software Corp, Cambridge, MA, USA). Temporal patterns of human cases and bovine shedding were then examined separately using the exact chi-square test (SAS v9.3.1, SAS Institute Inc., Cary, NC). Further analysis was conducted on PT21/28 and PT32 to compare the relative ratio of the two phage types in bovine isolates and human cases. If PT21/28 is associated with super-shedders (which are suspected to be linked to higher transmission rates) we should see high proportions in both cattle and humans whereas PT32 (associated with non super-shedders and potentially lower transmission rates) should be relatively over-represented in cattle.

## Results

### Animal Studies

14,849 faecal pats across 952 farms were sampled in the SEERAD study and 12,963 pats across 481 farms in the IPRAVE study. A total of 1,296 *E. coli *O157 strains were isolated from the SEERAD study (n = 207 farms) and 516 strains in the IPRAVE study (n = 91 farms). The spatial distribution of positive farms in the SEERAD and IPRAVE study are shown in Figure 1.

Among strains isolated during the SEERAD study, 0.2% (3/1231), 94.9% (1168/1231) and 4.9% (60/1231) possessed genes encoding the virulence factors *vtx*_*1 *_only, *vtx*_*2 *_only and *vtx*_*1*_*vtx*_*2 *_respectively. Among strains isolated during the IPRAVE study, 0.8% (4/508), 89.6% (455/508) and 8.9% (45/508) possessed genes encoding *vtx*_*1 *_only, *vtx*_*2 *_only and *vtx*_*1*_*vtx*_*2 *_respectively. All strains isolated from both studies possessed *eae*, the gene encoding the virulence factor intimin.

Farm and pat-level mean prevalence estimates for the two surveys are given in Tables 1 and 2 respectively. The point-estimate and confidence interval of group prevalence are both slightly higher than the raw estimates given earlier [28,34] as the figures now average over unbalanced random effects from the studies. Mean overall farm-level mean prevalence decreased slightly from 0.218 to 0.205 but this was not statistically significant (Table 1). Similarly, there was no significant change in temporal, seasonal or phage specific shedding at the farm-level. Mean overall pat-level mean prevalence of *E. coli *O157 more than halved from 0.089 to 0.040 (*P *< 0.001) (Table 2). The farm-level sensitivity of the IPRAVE study was only marginally smaller, at 81.8%, than that of the SEERAD study (86.2%), the effect of larger mean sample sizes being outweighed by the lower pat-level prevalences seen in the IPRAVE study. Over the same period, there were statistically significant decreases in the mean prevalence of shedding in all seasons. The mean pat-level prevalence decline was highly statistically significant (*P *< 0.001) in the North East and Central AHDs. Statistically significant decreases were also observed in the Highland and South East AHDs (*P *= 0.034 and *P *= 0.030 respectively). Among the major most common phage types, there was a substantial decrease in the mean pat-level prevalence of PT21/28 shedding from 0.052 to 0.019 (*P *< 0.001). PT21/28 was the dominant phage type isolated in both studies, representing 56% of strains in the SEERAD study and 51% of strains in the IPRAVE study. A statistically significant decrease in mean pat-level prevalence was also observed for PT2 (0.013 to 0.004). Changes in the mean pat-level prevalence of PTs 8 and 32 were not statistically significant.

**Table 1:** Mean farm-level prevalence of bovine *E. coli *O157 shedding for the SEERAD (March 1998-May 2000) and IPRAVE (February 2002-February 2004) surveys.

Category	Mean Prevalence (lower, upper 95% confidence limits)		*P*-value
		
	SEERAD	IPRAVE	
***All categories***	0.218 (*0.141, 0.320*)	0.205 (*0.135, 0.296*)	0.831

***By season***			
Spring	0.222 (*0.144, 0.325*)	0.191 (*0.125, 0.279*)	0.614
Summer	0.230 (*0.150, 0.335*)	0.262 (*0.177, 0.367*)	0.637
Autumn	0.262 (*0.173, 0.375*)	0.231 (*0.154, 0.330*)	0.648
Winter	0.149 (*0.094, 0.229*)	0.130 (*0.082, 0.199*)	0.674

***By animal health district***			
Highland	0.153 (*0.096, 0.234*)	0.198 (*0.130, 0.289*)	0.396
North East	0.248 (*0.163, 0.359*)	0.199 (*0.130, 0.290*)	0.442
Central	0.249 (*0.164, 0.359*)	0.204 (*0.134, 0.296*)	0.480
South West	0.189 (*0.121, 0.283*)	0.261 (*0.177, 0.366*)	0.257
South East	0.189 (*0.166, 0.364*)	0.231 (*0.168, 0.354*)	0.374
Islands	0.171 (*0.108, 0.259*)	0.111 (*0.070, 0.172*)	0.197

***By phage type***			
PT2	0.033 (*0.002, 0.352*)	0.017 (*0.008, 0.034*)	0.857
PT8	0.011 (*0.006, 0.020*)	0.019 (*0.01, 0037*)	0.278
PT21/28	0.135 (*0.067, 0.252*)	0.124 (*0.066, 0.219*)	0.865
PT32	0.031 (*0.0021, 0.378*)	0.060 (*0.019, 0.176*)	0.779

**Table 2:** Mean pat-level prevalence of bovine *E. coli *O157 shedding for the SEERAD (March 1998-May 2000) and IPRAVE (February 2002-February 2004) surveys.

Category	Mean Prevalence (Lower, Upper 95% Confidence Limits)		*P*-value
		
	SEERAD	IPRAVE	
***All categories***	0.089 (*0.075, 0.105*)	0.040 (*0.028, 0.053*)	<0.001

***By season***			
Spring	0.104 (*0.084, 0.126*)	0.044 (*0.024,0.0 66*)	<0.001
Summer	0.084 (*0.053, 0.118*)	0.039 (*0.022, 0.058*)	0.018
Autumn	0.085 (*0.061, 0.110*)	0.045 (*0.024, 0.069*)	0.016
Winter	0.074 (*0.035, 0.107*)	0.030 (*0.011, 0.054*)	0.045

***By animal health district***			
Highland	0.094 (*0.044, 0.170*)	0.023 (*0.008,0.045*)	0.034
North East	0.114 (*0.075, 0.161*)	0.024 (*0.005, 0.050*)	<0.001
Central	0.093 (*0.068, 0.118*)	0.033 (*0.011, 0.058*)	<0.001
South West	0.051 (*0.030, 0.073*)	0.068 (*0.026, 0.133*)	0.550
South East	0.106 (*0.074, 0.139*)	0.054 (*0.022, 0.091*)	0.030
Islands	0.064 (*0.028, 0.108*)	0.042 (*0.013, 0.077*)	0.396

***By phage type***			
PT2	0.013 (*0.008, 0.019*)	0.004 (*0.001, 0.007*)	0.007
PT8	0.004 (*0.001, 0.007*)	0.004 (*0.000, 0.009*)	0.821
PT21/28	0.052 (*0.039, 0.067*)	0.019 (*0.012, 0.028*)	<0.001
PT32	0.010 (*0.006, 0.014*)	0.007 (*0.003, 0.011*)	0.262

**Figure 2:**
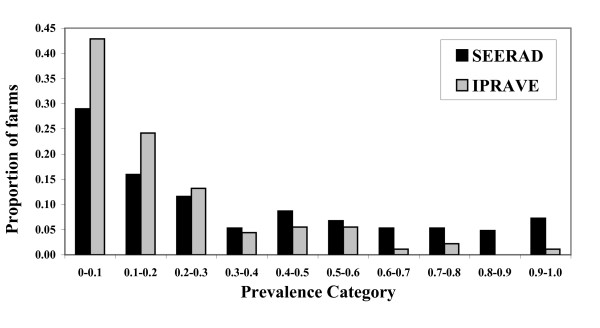
**Distribution of prevalence of *E. coli *serogroup O157 on positive farms**. Bars represent observed prevalence in faecal pats sampled from the SEERAD survey (black, n = 952 farms; n = 207 *E. coli *O157 positive) and IPRAVE survey (grey, n = 481 farms; n = 91 *E. coli *O157 positive).

In the majority of farms sampled in both surveys, no shedding animals were detected. The distribution of the prevalence on *E. coli *O157 positive farms is shown in Figure 2 for both the SEERAD and IPRAVE surveys. The distribution of prevalence for the two studies was different (Kolmogorov-Smirnov two-sample test: exact *P *< 0.001). The median prevalence of shedding animals was statistically significantly lower (Wilcoxon-Mann-Whitney test: exact *P *< 0.001) in the IPRAVE compared with the SEERAD survey (SEERAD: 0.25 (95%CI: 0.20-0.33); IPRAVE: 0.11 (95%CI: 0.09-0.14).

### Results from Human Data

Table 3 contains the number of culture positive, indigenous human *E. coli *O157 reported cases with known phage type results and the comparative equivalent incidence per year for the SEERAD and IPRAVE survey periods. There were 468 human cases between March 1998 and May 2000 (SEERAD) and 323 human cases between February 2002 and February 2004 (IPRAVE). The majority of reported human cases during each survey were PT21/28 with 320 (68% of total cases) and 232 (72% of total cases) total cases for the SEERAD and IPRAVE survey periods respectively. Declines were observed in the overall number of reported cases (468 compared with 323) and overall comparative annual incidence (215 compared with 161) as well as for all PTs with the exception of 'Other' PTs (Table 3).

**Table 3:** Culture positive indigenous human *E. coli *O157 cases with known phage-type results reported to HPS during the periods equivalent the SEERAD (March 1998-May 2000; n = 793 days; n = 468 cases) and IPRAVE surveys (February 2002-February 2004); n = 734 days; n = 323 cases).

Phage Type	Number of Cases		**Comparative Incidence**^**a**^ (Cases per Year)	
	
	SEERAD	IPRAVE	SEERAD	IPRAVE
All	468	323	215	161
PT2	51	23	23	11
PT21/28	320	232	147	115
PT32	22	7	10	3
PT4	19	9	9	4
PT8	31	22	14	11
'Other' PTs^b^	25	30	12	15

^a^Comparative incidence is equivalent to the number of cases per year. ^b^Includes PT34, PT14, PT31, PT33, PT54, RDNC and untypeable

### Comparison of Phage Types for Animal and Human Cases

The proportion of human cases and cattle isolates identified with *E. coli *O157 PT21/28 was much higher than any other phage type (Table 4). Overall there was a statistically significant association between time (SEERAD/IPRAVE) and PT for human cases and cattle isolates (CMH: 68.49, *P *< 0.0001). When human cases and cattle isolates were examined separately there were significant associations between time and PT although the associations for cattle isolates (exact χ^2 ^= 176.56, *P *< 0.001) were stronger than human cases (exact χ^2 ^= 11.75, *P *= 0.037). These results suggest that there was more temporal change in cattle isolates than in human cases.

**Table 4:** Comparison of the proportion of phage types between cases of culture positive indigenous human *E. coli *O157 cases with known phage type results reported to HPS and cattle isolates during the same periods of the SEERAD (March 1998-May 2000) and IPRAVE surveys (February 2002-February 2004).

Phage Type	Human Cases (Proportion)		Cattle Isolates (Proportion)	
	
	SEERAD	IPRAVE	SEERAD	IPRAVE
PT2	51 (0.109)	23 (0.071)	181 (0.147)	50 (0.098)
PT21/28	320 (0.634)	232 (0.718)	722 (0.587)	257 (0.504)
PT32	22 (0.047)	7 (0.022)	145 (0.118)	85 (0.167)
PT4	19 (0.041)	9 (0.028)	67 (0.0054)	6 (0.012)
PT8	31 (0.067)	22 (0.068)	56 (0.046)	51 (0.100)
'Other' PTs^a^	25 (0.053)	30 (0.093)	60 (0.049)	61 (0.120)

^a^Includes PT34, PT14, PT31, PT33, PT54, RDNC and untypeable

**Figure 3:**
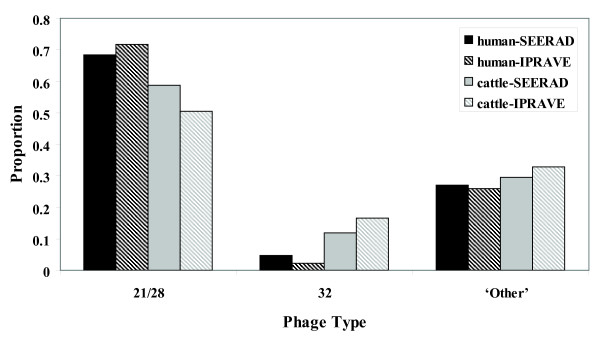
**Distribution of Phage types**. Proportion of Phage type (PT) 21/28, PT32 and 'Other' PTs in cattle isolates and in culture positive, indigenous human *E. coli *O157 cases with known phage type results reported to HPS, over the time periods equivalent to the SEERAD (March 1998 - May 2000) and IPRAVE (February 2002 - February 2004) surveys.

Figure 3 shows the proportion of PT21/28, PT32 and 'Other' PTs for human cases and cattle isolates collected during the SEERAD and IPRAVE surveys. PT21/28 was frequently observed in both human cases and bovine isolates. PT32 was more frequently observed in bovine isolates for both time periods. The relative ratio of the proportion of PT32:proportion of PT21/28 in cattle to the proportion of PT32:proportion of PT21/28 in humans is 2.92 and 10.96 for the SEERAD and IPRAVE surveys respectively, confirming that relative to PT21/28, PT32 is more common in cattle than human cases of *E. coli *O157. Overall there was a statistically significant difference in the distribution of these PTs between human cases and bovine isolates over the 2 time scales (CMH: 71.07 *P *< 0.001). There was no significant change in PT21/28, PT32 or 'Other' PTs for humans cases (exact χ^2 ^= 3.73, *P *= 0.158) whereas there were significant changes across time for bovine isolates (exact χ^2 ^= 12.24, *P *= 0.002).

## Discussion

The surveys examined in this study represent the only reported systematic national surveys of bovine *E. coli *O157 shedding and present a valuable opportunity to examine changes in patterns of shedding and strain characteristics. Knowledge of bovine shedding is important as cattle represent a major risk factor both for human *E. coli *O157 infection, whether from contamination of food or water by bovine faeces, or from direct contact with cattle or their environments, and for transmission to other animals. This is of particular concern in Scotland which has consistently higher rates of human *E. coli *O157 cases than the rest of the United Kingdom, and other European and North American countries [31-33].

In most instances it is difficult to compare results from different prevalence studies as different study designs, sampling procedures and microbiological methods have been used. The use of similar sampling and identical laboratory methods in the SEERAD and IPRAVE studies allowed direct comparison of *E. coli *O157 prevalence estimates. Estimates of the prevalence of *E. coli *O157 from the SEERAD study have been published, but in this study the estimates were recalculated to accommodate differences in sampling design and changes in statistical methodology. The farm-level and pat-level mean prevalence calculated for the SEERAD survey was 0.228 (95%CI: 0.196-0.263) and 0.079 (95%CI: 0.065-0.096) respectively [28]. In this study the same quantities were recalculated to be 0.218 (95%CI: 0.141-0.32) and 0.089 (95%CI: 0.075-0.105). These minor differences are the result of using different statistical models. Pat-level mean prevalence estimates for the IPRAVE study were generated using a bootstrapping technique given the clustered nature of data collection and the zero-inflated nature of the resulting data. For comparability the SEERAD pat-level prevalence was recalculated using the same methodology.

An overall comparison of the mean prevalence of *E. coli *O157 shedding for the SEERAD and IPRAVE surveys indicated a statistically significant decline in the mean prevalence of *E. coli *O157 at the pat-level but no statistically significant change at the farm-level. Over the 4-year period between the surveys there was a substantial decrease in the mean proportion of cattle shedding *E. coli *O157 on farms. The mean pat-level prevalence of *E. coli *O157 more than halved from 0.089 to 0.040 between the two surveys. This result possibly reflects a change in on-farm transmission rate between the two surveys, although the effect of environmental conditions or survival outside the host cannot be eliminated as possible causes of the differences observed. In two separate publications [35,36], the R_0 _(the average number of secondary cases generated by a single infected individual introduced into a naive population) of the SEERAD and IPRAVE surveys were reported as 1.9 [35] and 1.5 [36] respectively. A difference in transmission dynamics could explain the different distribution of prevalences observed in Figure 2. Higher transmission on a farm has been linked to the presence of super-shedding or high-level shedding animals [35,36]. As part of the IPRAVE survey, counts of *E. coli *O157 in pat samples were estimated. Unfortunately there is no data from the SEERAD survey on the density of *E. coli *O157 in farm pat samples. Therefore, no direct comparison between the numbers of super-shedders can be made between the two surveys. Research has shown that there is an association between the presence of a super-shedder and the presence of PT21/28 on a farm [37,42]. Therefore, we might hypothesise that there were fewer super-shedders on farms in the IPRAVE survey as opposed to the SEERAD survey as there were significantly fewer PT21/28 strains isolated in the IPRAVE survey. Assuming an association between shedding rates and transmission rates (R_0_) [39], fewer super-shedders may explain lower transmission rates on farms in the IPRAVE study and hence the lower mean on-farm prevalence. Unfortunately, in the absence of enumeration data from the SEERAD study this supposition cannot be tested.

Mean prevalence was calculated for different seasons, animal health districts (AHD) and phage types (PT). As observed with the overall prevalence results, statistically significant declines in mean prevalence of *E. coli *O157 were observed at the pat-level only. Marginal changes were observed at the farm-level but these were not statistically significant. The decline in the mean prevalence of pat-level shedding appears to have been driven by statistically significant reductions in the mean prevalence of PT21/28 as well as specific seasonal (spring) and regional (North East and Central) decreases.

Despite the statistically significant pairwise reductions in mean pat-level prevalences there was no equivalent change in overall mean prevalence at the farm-level. The mean farm-level prevalence between surveys did decline but it was not statistically significant. Changes in sampling strategy between the two surveys had a negligible effect on the power to identify positive farms, with the only potential effect of these changes being to reduce further the absolute size of the change in mean farm-level prevalences across the surveys. Dissociation between the mean prevalence at the pat and farm-level has been described for non-O157 strains of *E. coli *[51]. It is possible that at farm-level, *E. coli *O157 shedding may stop or remain undetectable in many cattle but still remain on the farm, and there are reports of extended *E. coli *O157 activity on individual farms [52]. This point has important implications for control programmes and assessment of their efficacy. Is it reasonable to conjecture that reductions in farm-level prevalence lag behind pat-level prevalence? Do we need to see more significant reductions in pat shedding over longer time periods before we might see a significant impact at the farm-level? Is this the result of bacteria maintained within the environment re-infecting cattle, or of a few persistently shedding cattle that are shedding at detectable levels but not transmitting to the rest of the group? Low-level shedders may have different risk factors but could have an important role in the maintenance of *E. coli *O157 populations on farms.

Sustained farm-level prevalence indicates persistence of *E. coli *O157 on farms, but decreases at the pat-level imply a lower environmental load which would expect to lower the force of infection to both cattle and humans. Concurrent declines in the total number and comparative annual incidence of human cases in this survey may reflect a link between human infection and the level of bovine shedding on a farm. However, the drivers of *E. coli *O157 infection are likely to be multifactorial, and as the infectious dose for *E. coli *O157 is low [53], a substantial reduction in environmental load may therefore be required to significantly reduce the risk of infection for humans.

PT21/28 is of particular concern because of its association with more severe human disease [41]. Analysis of human *E. coli *O157 cases over the same period as this study show that although it remains the dominant phage type, the incidence of phage type PT21/28 *E. coli *cases in humans declined [29] as did the prevalence of bovine shedding, providing circumstantial evidence of a link between bovine shedding and human infection. Our findings show that the relative ratio of PT32:PT21/28 in cattle pats compared with PT32:PT21/28 in human *E. coli *O157 cases was 2.92 during the course of the SEERAD study and 10.96 during the IPRAVE study. This supports the contention that phage type PT21/28 is more transmissible from cattle to humans than phage type PT32. The relative proportion of PT32:PT21/28 in cattle pats compared to human cases was much higher for the IPRAVE survey period, a reflection of the significant temporal change in the proportion of PT32 and PT21/28 in cattle between the two survey periods. The decrease in the proportion of PT21/28 and increase in PT32 were not mirrored by data on the human cases. Such results may be a reflection of the proposed heterogeneity in transmission [39]. In addition, PT32 may either be less stable in the environment than PT21/28 and/or less virulent to humans [41].

In this paper we have highlighted the importance of cattle as the primary source of human *E. coli *O157 infection. Cattle are the major reservoirs of *E. coli *O157 [54], they carry it asymptomatically in their intestines and excrete it in their faeces. Excretion rates for some animals (i.e. super-shedders) can be high (≥10^4 ^colony forming units (CFU) per gram of faeces) [34]. The potentially high excretion rate, longevity of *E. coli *O157 in pasture and soil [55] and the low infectious dose for human infection [53] mean that the environment is an important source of infection for humans. Comparison of 90 published *E. coli *O157 outbreaks meeting certain criteria (eg secondary cases were identifiable) from 9 countries [56] has identified exposure to contaminated food (54%) and environmental sources (including animal contact and water contamination) (17%) as the most frequently reported primary modes of transmission [56]. Analysis of general outbreaks (ie outbreaks involving the members of more than one household, or of institutions) of *E. coli *O157 infection in Scotland associated with either meat or dairy foods, or with environmental transmission (including direct contact with animals and their faeces and contaminated water supplies) showed that approximately 40% of these outbreaks were associated with foodborne transmission, 54% with environmental transmission and 6% with both modes of transmission [57]. However, most infections in Scotland are sporadic or single household cases, and not part of general outbreaks. Contact with livestock faeces was the risk factor most strongly associated with sporadic infection [10]. This further highlights the cattle and the environment as an important sources of *E. coli *O157 infections in humans.

**Figure 4:**
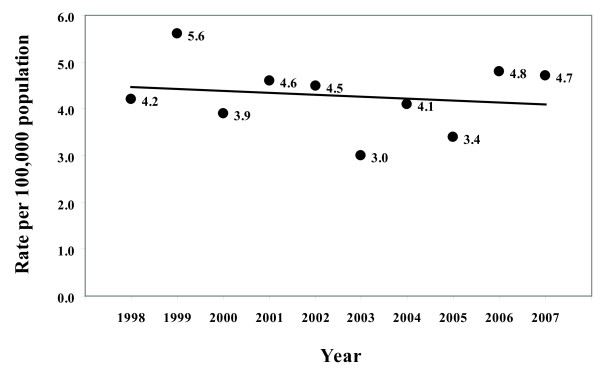
**Reported human *E. coli *O157 infections**. Rate per 100,000 population of all culture positive human *E. coli *O157 infections reported to Health Protection Scotland1998 to 2007. Source: Health protection Scotland. http://www.documents.hps.scot.nhs.uk/giz/graphs/2008/rates.pdf.

It remains to be seen whether the decline in the mean prevalence of *E. coli *O157 cattle shedding observed between the SEERAD and IPRAVE surveys continues, but there are precedents among other members of the Enterobacteriaceae family e.g. Salmonella [58] to suggest that this is possible. Despite observing declines in the number of human *E. coli *O157 cases over the time periods equivalent to the two cattle surveys, incidence rates, at least from 1998, do not seem to suggest a downward trend (Figure 4). Although these data were not generated by our study, examination of the reported rate of *E. coli *O157 infection per 100,000 population in Scotland shows that from 1998 to 2007 there was no change in the reported national rate of human cases (slope not significantly different from zero, *P *= 0.65) (Figure 4). Between 1998 and 2007 the average annual rate (per 100,000 population) was 4.28 (95% CI: 3.75-4.81) [30]. The IPRAVE survey included the year 2003, a year which had the lowest reported rate of human cases in Scotland since the early 1990s [30], suggesting that 2003 may have been an unusual year. In some regions of Scotland, 2003 was characterised by the highest temperatures and lowest rain fall since 1959 [59], and in the Islands, Highlands, and North East AHDs, the mean prevalence of *E. coli *O157 shedding in cattle was much lower in 2003 compared with 2002. Without linked data on the prevalence of bovine *E. coli *O157 shedding and the incidence of human cases over a longer time period, and more detailed linkage of geographical, temporal and meteorological data, the possible effects of climate must remain as conjecture.

## Conclusion

The objectives of this study were to assess the prevalence of bovine *E. coli *O157 shedding in Scotland; determine changes in the temporal, spatial and phage patterns of bovine shedding between the periods 1998-2000 and 2002-2004; and compare the phage types of *E. coli *O157 associated with human infections with those shed by cattle. Between the two survey periods, farm-level prevalence of shedding changed little, yet pat-level prevalence of shedding halved. This study also demonstrated that season, location and phage type are linked to pat-level prevalence of shedding. Between the two survey periods, human *E. coli *O157 case numbers also declined and the pattern of phage types shed by cattle were comparable to those isolated from human patients suggesting a link between bovine shedding and human infection. Our findings reinforce the need to reduce the prevalence and virulence of *E. coli *O157 shed by cattle in Scotland and the health risk posed by this organism [60,61].

## Authors' contributions

MCP collected farm data, analysed and interpreted data and prepared the manuscript. MECT analysed data and prepared the manuscript. IJM specified analyses and interpreted data. DJM and HET collected the farm data and interpreted data. LA, HIK and AWS conducted the laboratory analysis. MEL collected, applied inclusion criteria to, and provided the human data and contributed to the manuscript; WR authorised use of the human data. LM interpreted data and prepared the manuscript. MEJW, SWJR, BAS, JCL and GG supervised the study and interpreted data. All authors read and approved the final manuscript.
